# Time to take health economics seriously—medical education in the United Kingdom

**DOI:** 10.1007/s40037-015-0238-0

**Published:** 2016-01-07

**Authors:** Vageesh Jain

**Affiliations:** King’s College London, Guy’s Campus, Great Maze Pond, SE1 9RT London, UK

**Keywords:** Health economics, Medicine, Education, Curriculum

## Abstract

In the UK, the General Medical Council clearly stipulates that upon completion of training, medical students should be able to discuss the principles underlying the development of health and health service policy, including issues relating to health economics. With the National Health Service facing the threat of large gaps in funding, there is pressure on doctors to identify where and how savings can be made. Whilst many may be keen to learn about health economics, the teaching environment and level of student knowledge differs considerably across medical schools in the UK. There is a compelling argument to suggest that key concepts such as economic evaluation, equity and priority-setting should form part of the curriculum in UK medical schools. To address the complex nature of modern health care problems, doctors must have a perspective that combines medical expertise with economic proficiency.

On returning to medical school after studying a Master’s in Public Health, one aspect of the medical curriculum stands out to me, or rather doesn’t stand out: Health economics. Many of my classmates lack an understanding of how economics relates to health care. Some may not have been taught what a quality-adjusted life year (QALY) [1] is, and how it is used to prioritize treatments on the National Health Service (NHS). Others may be unable to contribute to discussions on the difficulties of financing health care in different systems across the world. This scarcity of economic knowledge directly contradicts the current culture in medical education. Young medical professionals and students are encouraged to have an impact on the system which they are a part of, to undertake audits and research, to improve clinical practice. It is illogical to pursue such a curriculum, whilst largely ignoring one of the most important aspects of modern health care: economics.

## What is health economics?

A relatively new field of practice, health economics uses economic theory to maximize the use of scarce resources, in turn improving the quality of health care and promoting evidence-based medical practice. The intention is to facilitate decision-making in a consistent manner, by offering an explicit framework based on the principle of efficiency.

One of the most central topics in health economics is economic evaluation. These evaluations offer a framework for measuring, valuing, and comparing the costs and benefits of different health care interventions. Such practice forms the basis behind whether one treatment or service is available over another. Another key concern of health economics is equity, which relates to priority-setting. Which treatments or services should be chosen over others, and who should benefit from them? With the advent of novel diagnostics and treatments, as well as rigorous ethical standards, these issues are becoming ever more vital to consider.

## Why is health economics important?

An understanding of cost-utility and cost-effectiveness analyses is essential if practitioners are to recognize the limitations of economic evaluations and use their clinical expertise in conjunction with national guidelines to provide the best possible care [2]. Equity and priority-setting questions form the basis of much debate in health economics as well as medicine. Yet, too few students have the knowledge needed to approach these complex challenges. Whilst many may be keen to learn about health economics, the teaching environment and level of student knowledge differs considerably across medical schools in the UK [3].

In the UK, the General Medical Council clearly stipulates that upon completion of training, medical students should be able to discuss the principles underlying the development of health and health service policy, including issues relating to health economics [4]. General practitioners (GPs) and other clinicians are often responsible for commissioning health care services, ensuring that outcomes are achieved whilst maintaining value for money. It is estimated that up to 50 % of the training places offered to medical graduates will be in general practice within the next few years [5]. Chronic illness and a nationwide shift to investment in community care means GPs are becoming increasingly important in today’s NHS. Yet, many are not equipped with the full range of necessary skills. It has been advocated that with increased financial responsibility, doctors require training in the economics inherent in health care to understand the implications of economic policy, guidelines and evidence-based practice [6]. In other countries such as the US and Germany, it has been proposed that providing high-value, cost-conscious care is to become a core competency for training doctors [7].

The role of economic practice in modern health care is becoming increasingly critical. Yet, there is a shortage of health economics expertise in the UK [8]. This is particularly worrying in current times of heated debate about instability and inefficiency within the NHS. Political expectations are growing that hospital doctors will get to grips with finances and be more involved in budget management and commissioning. But for many doctors a role in managing budgets or improving efficiency seems an overwhelming task, without much background knowledge, or training.

## The solution

With a rapid change in the political landscape, increasing pressure on the NHS and a need for those in top positions to influence the way health care is delivered, health economics needs to be effectively incorporated into the medical curriculum. If medical graduates are to improve the health systems in which they practice, they must be supplied the tools required to address problems of equity, cost and efficiency.

Since 2005 it has been mandatory for the NHS to provide treatments recommended by the National Institute for Health and Care Excellence (NICE) [9]. It can be argued that the evolution of evidence-based medicine and the role of NICE should be included in the UK medical curriculum. Other key concepts such as economic evaluation, equity and priority-setting could form part of the curriculum on health economics in medical schools. But before reaching such conclusions, a meaningful debate on the need, content and delivery of such a curriculum is essential.

A systematic needs assessment is first required to initiate improvement of the existing curriculum. Medical educators must then define a set of core competencies related to health economics, which will address existing gaps in need. The discussion about how best to deliver such a curriculum must involve key stakeholders including doctors, students and those working in health economics. Some may advocate the re-development of the curriculum to incorporate health economics throughout medical school, for instance into scenario-based teaching. Others may prefer the idea of expanding on existing educational outcomes, meaning health economics may fall under broader schemes such as systems-based practice, defined as a core medical competency by the Accreditation Council for Graduate Medical Education (ACGME) [10]. Systems-based practice requires that physicians understand how patient care relates to the health care system as a whole and how to use the system to improve the quality and safety of patient care.

An understanding of key concepts in health economics will allow doctors to understand and partake in the crucial policy debates relating to health care. To address the complex nature of modern health care problems, doctors must have a perspective that combines medical expertise with economic proficiency. Medical ethics and communication skills have become an integral part of the medical curriculum in recent years. Similarly, health economics must be considered fundamental to medical education, if we are to encourage an ethos of open-minded, progressive thinking to tackle the challenges of health care in the modern world.
